# Non-Contact Detection of Breathing Using a Microwave Sensor

**DOI:** 10.3390/s90402574

**Published:** 2009-04-14

**Authors:** Devis Dei, Gilberto Grazzini, Guido Luzi, Massimiliano Pieraccini, Carlo Atzeni, Sergio Boncinelli, Gianna Camiciottoli, Walter Castellani, Massimo Marsili, Juri Lo Dico

**Affiliations:** 1 Department of Electronics and Telecommunications, University of Florence, Via Santa Marta 3, 50139 Firenze, Italy; E-Mails: gilberto.grazzini@unifi.it (G.G.); guido.luzi@unifi.it (G.L.); massimiliano.pieraccini@unifi.it (M.P.); carlo.atzeni@unifi.it (C.A.); 2 Department of Medical and Surgical Critical Area, University of Florence, Viale Morgagni 85, 50134 Firenze, Italy; E-Mails: boncinelli@unifi.it (S.B.); mmarsili@unifi.it (M.M.); jr.ldc@alice.it (J.L.-D.); 3 Unit of Respiratory Physiopathology, University of Florence, Viale Morgagni 85, 50134 Firenze, Italy; E-Mails: g.camiciottoli@dac.unifi.it (G.C.); walter.castellani@unifi.it (W.C.)

**Keywords:** Microwave sensor, respiratory movement, vital signs, tidal volume

## Abstract

In this paper the use of a continuous-wave microwave sensor as a non-contact tool for quantitative measurement of respiratory tidal volume has been evaluated by experimentation in seventeen healthy volunteers. The sensor working principle is reported and several causes that can affect its response are analyzed. A suitable data processing has been devised able to reject the majority of breath measurements taken under non suitable conditions. Furthermore, a relationship between microwave sensor measurements and volume inspired and expired at quiet breathing (tidal volume) has been found.

## 1. Introduction

Non-contact microwave sensors for sensing breathing and heartbeat have been proposed since the early 1970s [1]. Recently interest in these sensors is rapidly increasing. State-of-art advances in the field of microwave technology have made it possible to design very small and simple devices for detecting vital signs [2–4]. New applications of non-contact sensing of human beings have also been conceived. While the early devices were mainly focused on medical applications, vital signs detectors have recently been proposed as law enforcement tools for detecting human beings hidden behind walls [5], or as rescue tools for finding survivors trapped under rubble [6] or under snow cover [7]. These applications have increased the interest in the potentiality of these kinds of sensors.

The research community is currently focussed on the use of two different technique for vital signs detection by means of microwave sensors: continuous wave (CW) radars [2,3,9,10] and ultra-wideband (UWB) radars [4,8].

In this paper a CW single frequency microwave sensor is described and employed to detect the signal backscattered by breathing movements. While most of previously published papers were focused on the detection of movement due to breathing or heartbeat in order to estimate their rate, this work is aimed at evaluating the use of this non-contact sensor for quantitative estimate of tidal lung volume.

Several CW radar configurations have been developed to deal with sensitivity and detection capability. C-band [9] and Ka-band [10] sensors have been tested to adapt the transmitted wavelength at the actual chest movements. At the same time, quadrature direct-conversion systems [2] or double sideband indirect-conversion systems [3] have been carried out to resolve the null point problem, which causes an accuracy decrease related to the distance between the sensor and the chest. However, all the above-mentioned systems involve non linearity into the measured output baseband signal.

The application proposed in this paper needs to measure the effective displacement of the chest during regular breathing, in order to obtain a relationship with the tidal volume. This goal can be reached by avoiding the harmonic and the intermodulation distortions, that are generated by systems in [2,3,9,10], by means of the interferometric technique, which uses the phase variation of the received microwave signal to calculate the displacement of the target. Therefore an S-band CW radar based on a quadrature heterodyne architecture has been fabricated and employed. Moreover, the static clutter must be cancelled, as shown in the next Section.

UWB radars transmit short pulses with pulse duration of the order of nanoseconds. These type of radars, as well as CW radars, are able to detect the movement of a target by measuring the low-Doppler variation that affect the received backscattered signal [4]. UWB radars also provide a range resolution that permits to eliminate the interfering pulses due to reflections of other targets in the field of view. However, that characteristic requires a fast switching time discriminator that open the receiver when the wanted reflected pulse arrives on it. If the distance changes, the delay of the time window of the discriminator must be changed.

CW radars are more simple systems than the UWB radars and the receiver is independent by the target distance. But in order to measure the displacements due to breathing, a particular attention must be taken: other movements of the subject under observation, different from that respiratory, should be avoided for both of the technique. Finally, supposing an application in clinical environment with more than one person in the cone of view of the antennas, the movement of other person different from that monitored degrades the measurement with CW radar, whereas it is irrelevant with UWB radar if it can be discriminated by the range resolution.

## 2. The Microwave Sensor

The sensor is basically a microwave coherent transceiver that irradiates a monochromatic wave in the field of view covered by two directional antennas, consisting of two four-elements patch arrays with about 20 degrees Half Power Beam Width.

The transmitter is a PLL (Phase Locked Loop) synthesizer with a 2.42 GHz carrier frequency: this operating frequency is in ISM (Industrial Scientific Medical) band and its use does not need a specific licence.

The receiver, based on a heterodyne architecture, detects the in-phase (I) and quadrature (Q) components of the backscattered field. The received signal is the coherent summation of the contributions from each scatterer included in the sensor field of view and also from possible multiple paths. This signal can be represented in the I–Q plane as a generic phasor, whose amplitude and phase are sensitive to movement as small as a fraction of wavelength.

The sensor is provided of a hardware clutter suppressor able to cancel the backscattered signal when in operation. The canceller works by injecting in the receiver chain a signal that is in opposition of phase to the received signal: an original algorithm performs an iterative search for the signal to be used for the cancellation [11]. As static component is generally greater than the informative dynamic component, the aim of the canceller is to prevent the saturation of the receiver caused by the non-informative static clutter.

The receiver performance has been measured on a breadboard arrangement: the I–Q amplitude mismatch was about 0.25 dB; the quadrature phase mismatch was about 0.5°; the second harmonic distortion was −50 dBc, and the third harmonic distortion was smaller than −60 dBc.

The proposed technique provides the presence of only one person in the cone of view of the radar, which is motionless except for the respiratory movement. In case of signals backscattered by two or more people that move themselves, the sensor detects a composition of their movement and is not be able to separate them. This aspect is a weakness of the CW radars, even if in literature attempts to resolve this problem can be found, by placing more than one sensor and so using spatial diversity [12].

In order to focus the sensor working principle, it is useful to take into account a simplified scenario with a single scatterer in motion along the sensor line of sight and in presence of static clutter.

**Figure 1.**
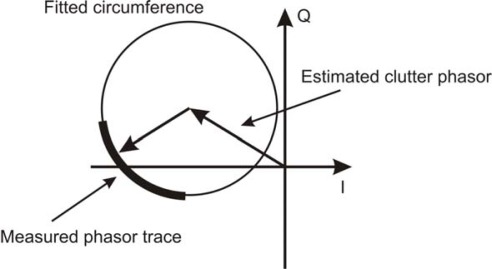
Phasor trace in I–Q plane due to static clutter and a rigid shift of a scatterer.

The static clutter is the summation of all contributions due to any kind of static objects, i.e. a barrier between the sensor and the body, objects like chairs or furniture or wall near the body, or also parts of the body that are not moving during the measurement. In the I–Q plane, the static clutter gives a static phasor, whereas the moving scatterer gives a phasor with a rotation Δ*φ* that is related to the displacement Δ*s* by the following simple relationship:

(1) $$\Delta \varphi =\frac{4\pi }{\lambda }\Delta s$$

with *λ* wavelength of the transmitted microwave. The graph in Figure 1 shows in the I–Q plane the combination of the phasor of a static clutter and that of the shift of a single scatterer, without multipaths. The phasor describes an arc of circumference. Therefore, the phasor of the static clutter can be removed simply by finding the centre of the circumference that best fits the measured phasor trace. An effective algorithm for this operation is the nonlinear minimum square Levenberg-Marquardt method [13,14] with a parameterization proposed by Chernov-Lesort [15]. The search of the fitting circumference is made easier by a trace covering a great angle with small dispersion. It should be noted that the static clutter removal is essential in order to correctly detect the movements: indeed the presence of the static clutter affects not only the amplitude of the movement retrieved through the phase shift, but also the qualitative temporal behaviour of the signal.

Using the described circle fitting method to isolate the movement information, the microwave sensor is able to measure displacements of a scatterer along the line of sight of the antennas. The moving targets for this application are the trunk, the thorax and the abdomen of a human body. The respiratory movements depend on the combined action of different muscle systems, that produce both a posterior to anterior expansion and a smaller lateral expansion of the chest and the abdomen [16].

Even though the main contribution to the echo signal is the reflection of the first superficial layer rather than the contribution of the inner layers, the trunk of a human body is a complex electromagnetic target, which is not possible to characterize with simplified models.

However, some general remarks can be made.

If the microwave sensor is positioned in front of the chest, the target has an approximately symmetric geometry. Moreover, by assuming the frontal displacement of the target predominant, for regular respiration with thoracic and abdominal coherent movements, the backscattered signal in the I–Q plane should describe a circumference arc as in the single scatterer case. In these conditions, the displacement due to respiration can be estimated by using the method described above. Obviously all these considerations must be verified through experimental measurements, as discussed in Section 4.

In normal subjects the respiration movements at rest are small enough that volume variation of air breathed in and out can be assumed in linear relationship with the frontal displacement of the trunk of the body. Also this assumption must be experimentally demonstrated (see Section 4).

The linear relationship between frontal displacement and tidal volume can be used to evaluate the volume itself, provided a suitable calibration. The conventional clinical tool for measuring the tidal volume is the spirometer. In order to find the relationship between microwave measurement and volume variation, a simultaneous acquisition with both the microwave sensor and the spirometer must be carried out.

## 3. Quality Index of the Measurement

As above mentioned, in ideal conditions the phasor of a measurement describes an arc of circumference in I–Q plain. Unfortunately in practical cases, measurements can generate distorted arcs due to the particular pattern of breathing, i.e. by mixing abdominal and thorax not in-phase movements, or to the movement of other parts of the body, i.e. shifting of the head or the shoulders.

**Figure 2.**
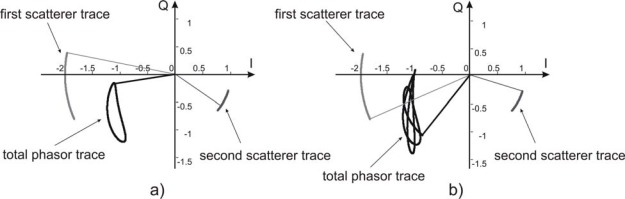
Phasor traces of the simulated signal due to two scatterers at distance 1.02 m and 0.99 m, with a sinusoidal motion with 6mm and 4 mm peak-to-peak amplitude respectively. (a) oscillation at 0.25 Hz movement have a phase shift of *π*/3. (b) oscillation frequencies are 0.25 Hz e 0.30 Hz respectively.

Such irregular movements are possible sources of error. Some simplified scenarios can help to focus the main causes of error: a) two scatterers harmonically moving with different phase shifts; b) two scatterers harmonically moving with different periods. Both these cases give phasors that do not lie on a circumference in I–Q plane, as illustrated in Figure 2, where the results of a simulation with 2.42 GHz transmitted carrier is shown. In particular Figure 2a shows the case of two scatterers at distance 1.02 m and 0.99 m from the sensor, moving both at 0.25 Hz with π/3 radians phase shift and a peak-to-peak amplitude of 6 mm and 4 mm respectively. Figure 2b shows the two scatterers as in the previous case, but oscillating at different frequencies: 0.25 Hz and 0.30 Hz respectively.

The presence of multi-path echoes can be an additional source of error. The presence of contributions due to different paths results in a phasor similar to that produced by irregular movements, as confirmed by several simulations. Unfortunately, although the phasor trace is not a circumference, for small multi-path displacement it can easily be confused with an arc of circumference, giving a possible error in the evaluation of the effective displacement of the moving target.

In practical cases, all these causes could produce measurements affected by large errors. For this reason a quality index to discriminate and reject not suitable measurements has been defined as follows.

**Figure 3.**
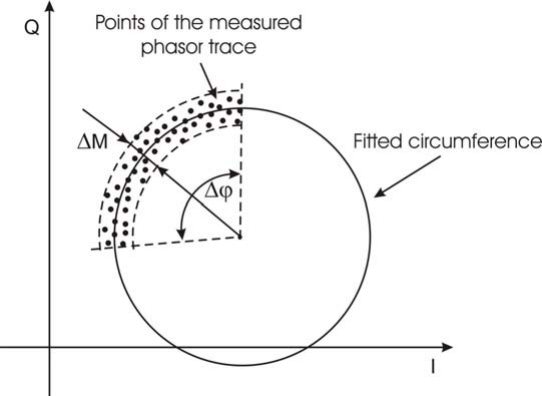
Representation of the dispersion of the measured phasor trace: Δ*M* is the dispersion along the radial direction of the circumference; Δ*φ* is the dispersion along the angle under the arc of circumference.

Let Δ*M* be the phasor dispersion along radial direction of the circumference, and Δ*φ* the dispersion along the angle under the arc of circumference, as shown in Figure 3. By multiplying Δ*φ* by circle radius, the dispersion Δ*C* along the circumference arc can be calculated. By assuming standard deviations *σ_M_* and *σ_C_* along the two directions as dispersion indexes, the following ratio:

(2) $$D=\frac{\sigma_{C}}{\sigma_{M}}$$

can be used for quality evaluation of the measurement: the greater is *D*, the better is the measurement.

## 4. Experimental Results

The experimentation has been carried out using the microwave sensor described above and a spirometer (model VMAX 22, SensorMedics, Yorba Linda, CA, USA). This is a heated wire sensor and needs a preliminary calibration before the use. The calibration is carried out using a 3 litre calibration syringe.

**Figure 4.**
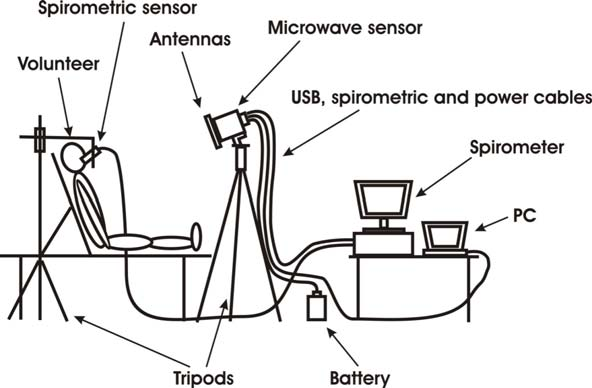
Sketch of the measurement set-up.

The experimental set-up was arranged in the Respiratory Physiopathology Section of the University of Florence, and it involved 17 volunteers (males and females) with age ranging from 26–76 years (Table 1). Each volunteer was laying on an examination couch with the back elevated; Figure 4 shows a scheme of the set-up. Head and shoulders were leaned against the couch.

Two measurements, each of 90 seconds, were acquired using the microwave sensor and spirometer simultaneously. The microwave sensor was positioned in front of the patient at about 1 m distance in such a way that the chest was approximately orthogonal to the microwave beam. The spirometric sensor was held close to the mouth by a tripod.

An acquisition unit acquired at 1 kHz sampling frequency simultaneously both the sensor signal and the spirometer data. As the microwave power was 10 mW and the antenna gain was 12 dB, the power density on the patient resulted 1.26 × 10^−2^ W/m^2^. This value fully respects the ICNIRP (International Commission Non-Ionizing Radiation Protection) guidelines [17].

After static clutter cancellation of the microwave signal using circle fitting method, both microwave and spirometric data were filtered with a 10 Hz low pass filter for reducing high frequency noise contribution.

The quality of each measurement was evaluated by calculating the ratio (2). On the basis of the experimental data the following empiric threshold has been selected:

(3) D≥7

This is an arbitrary value that accords with the experience in the analysis of this type of measurement. It permits the circle fitting algorithm to obtain a good agreement between the phasor trace and the fitted circumference, thus discarding the measurements in case of heavy non ideal conditions, as explained in Section 3.

**Figure 5.**
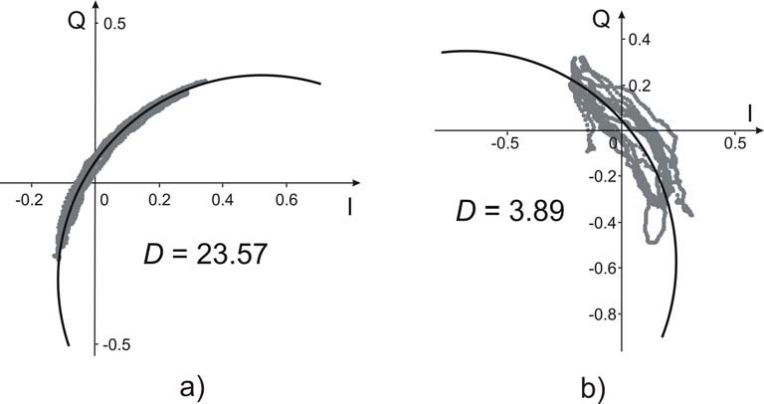
Experimental results in *I–Q* plane. Examples of two extreme cases: **(a)** valid data with *D* = 23.57; **(b)** discarded data with *D* = 3.89.

As examples of two very different cases, a valid measurement with *D* = 23.57 and a discarded measurement with *D* = 3.89 are shown in Figures 5a,b, respectively.

The two measurements of 90 seconds for each volunteer have been divided in intervals of 30 seconds, so for each volunteer six datasets have been analysed. The tidal volume was evaluated only for the 12 subjects which have collected at least four datasets with *D* ≥ 7 (Table 2).

**Figure 6.**
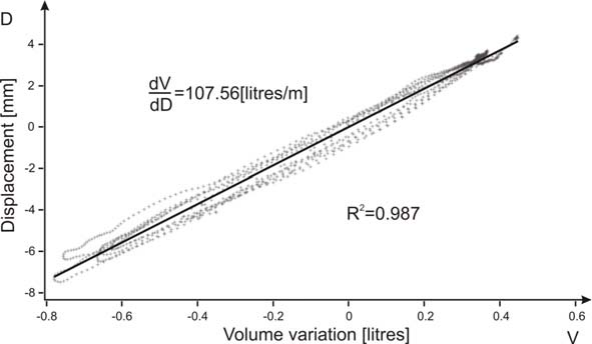
Displacements measured by the microwave sensor as a function of the volumes measured through the spirometer for one data set. Solid line represents the calculated linear regression.

The first 30 seconds dataset of the first measurement for each volunteer was used to verify the linear relationship between volume variation, measured by the spirometer, and displacement variation, measured by the microwave sensor. An example of the obtained linear regression for one dataset is shown in Figure 6. Table 3 summarizes the constants of proportionality of the 12 selected volunteers; it can be seen that the linear regression gives very high *R*^2^ coefficients, thus well verifying a linear relationship.

In order to estimate the tidal volume, the second measurements, divided in three series of 30 seconds, have been considered. 30 datasets for the test were selected according to condition (3). After calibration, the tidal volume versus time was measured using both microwave sensor and spirometer.

**Figure 7.**
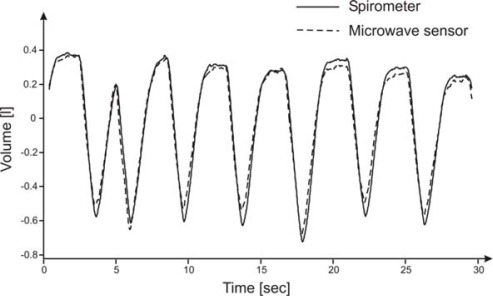
Volume variation measured by spirometer and by microwave sensor after calibration, as a function of time, for a single data set.

Tidal volume is the difference between maximum and minimum volume along the entire duration of the dataset: an experimental result is shown in Figure 7. The linear regression of the tidal volumes of the 30 datasets measured by the two different methods has got a slope close to 1 (0.986).

**Figure 8.**
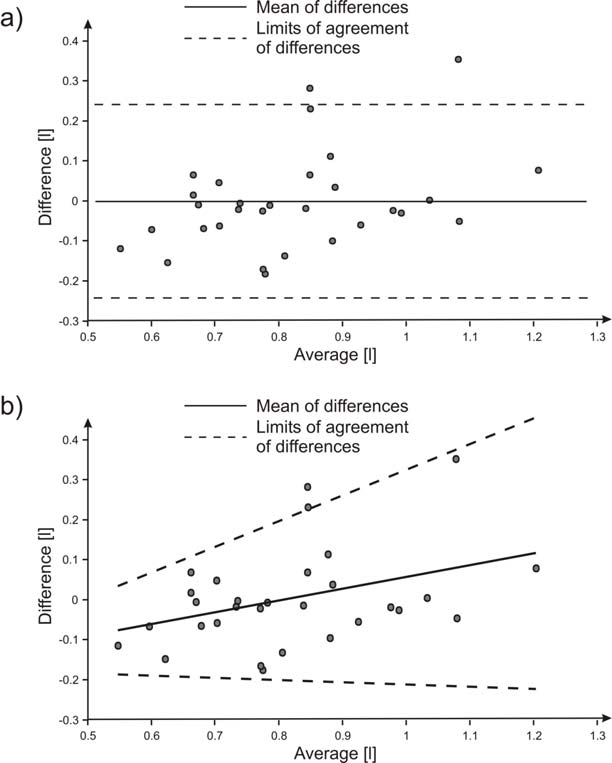
Differences versus average for tidal volumes measured with two different methods: (a) mean and 95% limits of agreement considering uniform dispersion; (b) mean and 95% limits of agreement with regression approach.

A correct approach to compare two different measurement methods has been proposed by Bland and Altman [18,19]. It consists in drawing a graph of the difference *D* between the values obtained through the two methods versus their average *A* (Figure 8a). The bias error (mean of differences) is *ε* = −0.001 litre and the measurements lie in the interval −0.001 ± 0.241 litre with a probability of 95% by supposing a Gaussian distribution.

The difference *D* of the points in Figure 8a, for low and high average *A* of tidal volume, exhibits a trend to increase with increasing volume, as well as a non-uniform dispersion around the line of best fit. Therefore, a better representation of the results is possible using a regression approach both for bias error and for dispersion of the differences [19]. Figure 8b shows the results where *ε* = −0.246 + 0.298 × *A* litre and 95% limits of agreement is −0.246 + 0.298 × *A* ± 2.46 × (−0.033 ± 0.143 × *A*) litre.

## 5. Conclusions

The aim of this work is a preliminary evaluation of the potentiality of an innovative non contact microwave sensors for the measurement of tidal lung volume. Comparison with the results provided by a conventional spirometer, obtained through experimentation on a group of volunteers, has demonstrated that, after calibration with a spirometer in the position of measurement, microwave sensor can achieve quantitative results. Accuracy of the tidal volume estimation, however, is not very high, and the new methodology need a preliminary calibration with a spirometer for every subject. Nevertheless, these encouraging results indicate new perspectives for monitoring thoracic movements and tidal volumes without contact.

## Figures and Tables

**Table 1.** Series of parameters associated to the experimental sample.

**Volunteer**	**Sex**	**Age**	**Weight [kg]**	**Height [cm]**	**Chest Circumference [cm]**
1	M	47	67	176	97
2	M	76	66	171	91
3	M	27	70	168	95
4	M	27	80	173	99
5	M	31	80	175	106
6	M	70	78	169	103
7	F	62	54	150	99
8	F	30	68	174	90
9	F	28	54	160	79
10	F	28	62	170	82
11	F	30	60	170	83
12	F	30	56	168	84
13	M	64	78	172	108
14	M	24	71	175	96
15	F	28	55	160	86
16	F	28	49	160	80
17	F	29	64	171	92

**Table 2.** Subdivision of volunteers based on quality index.

**Dataset with***D* ≥ 7	**Number of volunteers**		
0	2	5	**Number of rejected volunteers**
1	0		
2	2		
3	1		

4	2	12	**Number of selected volunteers**
5	2		
6	8		

**Table 3.** Constant of proportionality and coefficient of determination for every volunteer.

**Volunteer #**	**Constant of proportionality [L/m]**	**R^2^**
1	89.24	0.967
2	56.07	0.955
3	51.34	0.917
4	136.53	0.977
5	76.22	0.949
7	74.03	0.968
8	64.05	0.955
10	167.96	0.976
12	113.51	0.980
14	88.99	0.936
15	81.33	0.984
17	107.56	0.987
